# The Multiple Functions of Amyloid Beta in the Gut Epithelium and the Role of the Microbiota: A Study in the APP/PS1 Animal Model Subjected to Chronic Synbiotic Treatment

**DOI:** 10.3390/nu18121883

**Published:** 2026-06-11

**Authors:** Giorgia Sarti, Giorgio Tognozzi, Giada Magni, Daniele Lana, Francesca Rossi, Chiara Traini, Maria Giuliana Vannucchi

**Affiliations:** 1 Research Unit of Histology and Embryology, Department of Experimental and Clinical Medicine, University of Florence, 50134 Florence, Italy; giorgia.sarti@unifi.it (G.S.); giorgio.tognozzi@unifi.it (G.T.); chiara.traini@unifi.it (C.T.); 2 Institute of Applied Physics “Nello Carrara”, CNR, Sesto Fiorentino, 50019 Florence, Italy; g.magni@ifac.cnr.it (G.M.); f.rossi@ifac.cnr.it (F.R.); 3 Section of Clinical Pharmacology and Oncology, Department of Health Sciences, University of Florence, 50134 Florence, Italy; daniele.lana@unifi.it

**Keywords:** Alzheimer’s disease, colon, ileum, enterocytes, beta-amyloid, microbiota, APP/PS1 mice, intestinal barrier

## Abstract

**Background:/** Over the past decade, increasing evidence has shifted attention from the brain to the gut microbiota (MB) as a source and site of systemic dissemination of amyloid-β (Aβ), an APP derivative responsible for plaque formation in the brains of Alzheimer’s disease (AD) patients. Furthermore, AD patients and APP/PS1 mice, a transgenic model of AD, exhibit dysbiosis. **Objectives**: Using APP/PS1 mice treated from 2 to 8 months of age, we studied ileal and colonic epithelial integrity, intestinal barrier (IB) integrity assessed through tight junction (TJ) protein expression, local immune system, the presence/increase in Aβ expression in enterocytes, and the protective effects of synbiotic treatment. **Methods:** The tissue was stained with Periodic Acid-Schiff and Alcian Blue to evaluate epithelial morphology and mucus production, and immunohistochemistry was performed to assess TJs, immune markers, and Aβ expression. **Results:** Our results demonstrate that colonic and ileal epithelium of 8-month-old APP/PS1 mice displays IB impairment in term of alterations of goblet cells staining and TJ protein expression and signs of immune involvement. The ileum was more severely affected, showing a reduced epithelial surface area, decreased lysozyme production, and fewer tuft cells. Long-term synbiotic treatment largely prevented APP/PS1 mouse changes and caused a significant increase in Aβ expression in all treated mice. **Conclusions:** These findings support the belief in early intestinal involvement in AD and highlight the potential of the microbiota as a target for early intervention aimed at modifying the progression to neurodegeneration. Increased epithelial Aβ labeling after treatment raises the possibility of intestinal management of Aβ, which requires further validation.

## 1. Introduction

Deposits of amyloidogenic or prion-like proteins in the brain, around the capillaries, or in the parenchyma are typical histopathological signs of tissue damage accompanied by glia activation, neuroinflammation, and neuronal loss [1]. Many of these proteins have a peripheral origin, deriving either from the diet or from synbiotic microorganisms forming the gut microbiota (MB). In both cases, the access gate to the organism is the gut [2].

Amyloid beta (Aβ) is a cleavage product of the amyloid precursor protein (APP), which is found in cell membranes and is preferentially cleaved in the endosomal system [3].

Two distinct enzymes called β-secretase and γ-secretase (presenilin 1), which act sequentially, cleave APP at two different sites, producing a 37–49-amino-acid-long Aβ peptide [4]. The γ-secretase is responsible for the peptide length, making slightly different cuts at the *C*-terminal, which most commonly results in a peptide of 40 (Aβ1–40) or 42 (Aβ1–42) amino acids. APP and its derived peptides have a central role in the neuronal physiology, being essential in regulating synaptic function, axonal transport, and neuronal growth [1]. Thus, these cleavage products are not intrinsically pathogenic, and the disease arises from an abnormal folding state of Aβ/APP-derivative peptides [3].

In exploring the cellular mechanisms of AD, attention has most often been focused on the Aβ1–42 peptide because of its tendency to aggregate in vitro [5], its ability to stimulate tau protein hyperphosphorylation [6], and its pronounced neurotoxic effects [7]. Consequently, a histopathological correlation has been formulated between the occurrence of deposits of a fibrillar and insoluble form of Aβ1–42 in the brain and AD [8], and these deposits, organized in extracellular plaques, are considered the hallmark of this type of dementia [9]. This correlation, however, might overlook the complexity of Aβ1–42 pathophysiology, whose role is not limited to the presence of the peptide but depends on its aggregation state [10], the size of the resulting fibrils [11], and how mature fibrils change conformation over time [12]. This complexity is further increased by different Aβ1–40 and Aβ1–42 ratios, which generate heterogeneous fibril populations with distinct structural and seeding properties [13]. Together, these processes lead to the formation of heterogeneous plaques across individuals and pathological conditions [14,15,16].

This heterogeneity may help explain the repeated failure of therapies targeting cerebral Aβ deposits in clinical trials [1,17,18], suggesting that a brain-centric view of the disease may be insufficient. This has led to a shift in focus toward a possible peripheral source of Aβ and toward systems capable of regulating its deposition and clearance at a systemic level, such as the gut MB system within the gut–brain axis [19,20,21,22].

In dysbiosis, characterized by alteration of the MB composition, several microbes such as *Pseudomonas aeruginosa* and *Escherichia coli*, which are opportunistic pathogens, increase in quantity and produce fibrillar structures similar to Aβ amyloids [23]. This condition is associated with an increased intestinal barrier (IB) permeability, further contributing to systemic exposure to microbial and inflammatory signals [22,24]. Enterocytes have been shown to produce APP and Aβ peptides [25,26,27]. Furthermore, under certain conditions and through various mechanisms, viruses and bacteria can alter the production of Aβ and, above all, its aggregation state [28,29,30]. Moreover, plasma levels of soluble Aβ have been reported to correlate with brain Aβ burden in both healthy individuals and AD patients [25,31]. These findings support the concept of a systemic component in Aβ homeostasis, extending beyond the central nervous system (CNS).

In Alzheimer patients and in AD animal models, qualitative and quantitative changes in the MB composition have been reported [20,32,33,34,35], and this dysbiosis has been associated with IB damage [36] and intestinal innate immunity activation that may precede the onset of CNS neuroinflammation [37]. In addition, dysbiosis has been suggested to contribute to alterations in blood brain barrier (BBB) function [22,38,39], with an overload or defective clearance of Aβ in the CNS followed by Aβ accumulation, fibril organization, and plaque formation [39] Experimentally, fecal microbiota transplantation (FMT) from Alzheimer patients into mice has been shown to induce cognitive deficits and impair neurogenesis [40], supporting a causal role of the MB in AD-related phenotypes.

Despite all these published results, the real role of amyloidogenic peptides in the intestinal epithelium is poorly understood. An Australian research group showed a relationship between cholesterol, fatty acids, lipoproteins, and quantitative changes in Aβ expression in the ileal enterocytes, where Aβ has been implicated in lipid binding and transport from blood to brain [26,27,41]. Wu et al. hypothesized that the intestine is a site of Aβ excretion and a potential route for peripheral clearance of Aβ, emphasizing that Aβ1–42 clearance could be modulated by the MB composition [42]. These reports indicate that the intestinal Aβ may also have a physiological role in mediating communication along the gut–brain axis. Recently, Liu et al. reported the presence of Aβ in intestinal CD8-positive mucosal cells of AD transgenic mice, although its functional significance remains unclear [43].

In summary, available information proposes that targeting MB could improve both gut and brain symptoms in AD through the MB–gut–brain axis [44,45,46]. In this context, we previously demonstrated that long-term synbiotic treatment of APP/PS1 female and male mice improved cognitive performances, prevented changes in colonic mucus secretion and intestinal activity, modulated MB composition, and reduced Aβ deposition and microglia activation in the cortex and hippocampus [47,48,49].

Presently, in an APP/PS1 animal model treated as previously described [47,48,49], we investigated the ileal and colonic epithelium to assess (i) IB integrity and immune system in Tg mice; (ii) the effectiveness of prebiotics and probiotics to prevent these alterations; (iii) Aβ1–40/42 expression in enterocytes; (iv) the impact of synbiotic intervention on amyloidogenic peptides regulation. The choice to co-administer prebiotics and probiotics was based on limited but clear evidence in both animals and humans that co-treatments are more effective than single treatments. Particularly, we used a prebiotic blend rich in inulin and fructo-oligosaccharides and two species of lactobacilli that produce lactate and acetate and contribute to other short-chain fatty acids (SCFAs) production through cross-feeding mechanisms by metabolizing the two aforementioned molecules [50,51].

## 2. Materials and Methods

### 2.1. Animals and Treatments with Probiotics and Prebiotics

The mice were housed in the Laboratory Animal Housing Center (CeSAL) facility of the University of Florence, with 3/4 mice per cage, under standard laboratory conditions (room temperature: 21 ± 2 °C, 12:12 h light–dark cycle) with food and water available ad libitum. All experimental protocols were carried out in accordance with the European Communities Council Directive 2010/63/UE and approved by the Italian Ministry of Health (code: 53/2022) [47].

The number of animals per group varied from 6 to 10 mice, with an unbalanced distribution between the sexes since no sex-related differences had been observed in these same animals in our previous researches [47,48,49].

Two-month-old male and female APP/PS1 (Tg) mice and two-month-old male and female littermate mice (Wt) were randomly placed in one of the four following experimental groups: the Wt group fed with a standard diet; the Wt-T group fed with the standard diet enriched in prebiotics and probiotics; the Tg group fed with a standard diet; the Tg-T group fed with the standard diet enriched in prebiotics and probiotics [47].

The mice were treated over a six-month period, beginning at the end of the second month until 8 months of age [47]. Throughout the six-month treatment, no significant changes in food or water consumption were observed, indicating that the prebiotic- and probiotic-based diet did not affect the animals’ nutritional demands [47].

The probiotic consisted of a 50% mixture of each of the following bacteria: *Lactobacillus rhamnosus* IMC 501 and *Lactobacillus paracasei* IMC 502 daily administered at a dose of 1.8 × 10^8^ CFU/day/25 g per mouse. The probiotic was dispersed directly into gelled drinking water with instant corn-starch-based powder thickener. The untreated mice were supplied with gelled drinking water during the entire treatment period to maintain standardized experimental conditions for all groups.

The prebiotics added to the standard diet were a multi-extract of fibers and plant complexes, mainly composed of inulin/FOS (fructo-oligosaccharides). The prebiotics, added at the dose of 50 mg inulin/FOS/g diet, corresponding to a 5% increase in inulin/FOS, had identical organoleptic and morphological characteristics to the standard diet [47,48,49].

### 2.2. Sample Preparation

At the end of the eighth month, the mice were deeply anesthetized through the subcutaneous administration of ketamine–dexmedetomidine mixed solution (80–120 mg/kg + 0.5–1.0 mg/kg, respectively) and euthanized by guillotine. The intestine was removed and samples of the ileum and proximal colon were immediately fixed in 4% paraformaldehyde in 0.1 M phosphate-buffered saline (PBS, pH 7.4) overnight (ON) at 4 °C [47,48,49]. The samples were embedded in paraffin and sectioned using a microtome (HistoCore MULTICUT, Leica, Buccinasco, MI, Italy) to obtain cross sections 5 μm thick that were collected on normal or positively charged slides for histochemistry or immunohistochemistry studies, respectively.

### 2.3. Histochemistry

The uncharged slides were stained with Hematoxylin Eosin (H$E) to quantify the epithelium surface of the ileum; with Periodic Acid-Schiff reagent (PAS) to quantify the number of goblets cells and with Alcian Blue (A-Blue), at a pH of 2.5, for analyzing the acidic component of the mucus. At the end of each staining procedure, the slides were dehydrated, clarified, and mounted in synthetic resin. All sections were stained in a single session to minimize artefactual staining differences, and two sections per animal were analyzed [47,48,49].

### 2.4. Immunohistochemistry

Aβ1–40/42 single labeling: Ileum and proximal colon paraffin-embedded sections were deparaffinized in xylol for 40 min and in clean xylol for another 15 min and dipped 20 times in 50% ethanol/xylol. Subsequently, the sections were rehydrated by immersing them in gradually decreasing alcohol gradients (specifically 100%, 95%, 75%, and 50%) for 5 min each and then immersed in bi-distilled water for 5 min. The sections were treated for antigen retrieval for 15 min in boiling water with EDTA (1 mM, pH 8.0), without overboil, in the microwave (350 W), followed by cooling to RT. The sections were washed in PBS 1x for 5 min and then permeabilized for 15 min in PBS-Tween 0.2%. The sections were blocked for 30 min with 20% normal donkey serum (NDS), (Jackson Laboratories, Bar Harbor, ME, USA) in blocking buffer, BB, (BB: 50 mM glycine, 0.05% Tween20, 0.1% Triton X100, and 0.1% BSA diluted in PBS 1x) for blocking the non-specific staining. The primary antibody Aβ1–40/42, 1:200, (Chemicon, see Table 1) was diluted in 10% NDS in Antibody Signal Enhancer, ASE, (ASE: 10 mM glycine, 0.05% Tween20, 0.1% Triton, and 0.1% hydrogen peroxide) and incubated ON at 4 °C. The next day, the sections were washed 3x5 min in PBS-Tween 0.2% in fast agitation and then incubated for 1 h at room temperature in the dark with goat anti-rabbit Alexa Fluor 568, 1:100, (Jackson Immuno research, see Table 1) in 10% NDS diluted in PBS-Tween 0.2%. Tissue sections were thoroughly washed in PBS-Tween 0.2% in fast agitation 3 × 5 min and incubated with a solution of Hoechst, 1:1500, (Enzo Life Sciences, Farmingdale, NY, USA; code: 52401) in PBS-Tween20 for 10 min. The slices were washed in bi-distilled water 2 × 5 min and then mounted with an aqueous medium (FluoreGuard Mounting Medium, ScyTek Laboratories Inc., Logan, UT, USA) [47,48,49].

DCLK1 single labeling: Ileum and proximal colon paraffin-embedded sections were deparaffinized in xylol 2 × 5 min each and rehydrated by immersing them in gradually decreasing alcohol gradients (specifically 100%, 95%, 75%, and 50%) for 2 min each. Subsequently, the sections were immersed in bi-distilled water for 5 min. The sections were treated for antigen retrieval for 3 × 3 min cycles with 2 min rest between each cycle in boiling water with Citrate Buffer solution 1× pH 6.0, without overboil, in the microwave (90 W), followed by cooling to RT. The sections were washed in PBS 1x for 3 × 2 min and then were blocked for 20 min with 5% NDS (Jackson Laboratories, Inc.) in PBS 1x for blocking the non-specific staining. The primary antibody DCLK1, 1:100, (Abcam, see Table 1) was diluted in 1.5% NDS and incubated ON at 4 °C. The next day, the sections were washed 3 × 10 min in PBS 1x and then incubated for 2 h at RT in the dark with goat anti-rabbit Alexa Flour 568 (Invitrogen, see Table 1), pre-diluted 1:1 in 50% glycerol and used at a final dilution equivalent 1:333 in 1.5% NDS. Tissue sections were washed in PBS 1x for 3 × 10 min and were incubated with a solution of Hoechst 1:1500 (Enzo life sciences, code: 52401) in PBS 1x for 10 min. The slices were washed in bi-distilled water 2 × 5 min and then mounted with an aqueous medium (FluoreGuard Mounting Medium, ScyTek Laboratories, Inc.) [47,48,49].

Claudin-2, ZO-1, and Lysozyme single labeling: The ileum and proximal colon paraffin-embedded sections were deparaffinized in xylol 2 × 5 min each and then were rehydrated by immersing them in gradually decreasing alcohol gradients (specifically 100%, 95%, 75%, and 50%) for 3 min each. Subsequently, the sections were immersed in bi-distilled water for 5 min. For antigen retrieval, the sections were warmed for 20 min at 92 °C with Basic Buffer (Tris buffer 10 mM added with EDTA 1 mM at Ph 9.0). The slices were then cooled to RT, washed in PBS 1x, 3 times, 2 min each time, and blocked with 5% NDS (Jackson Laboratories, Inc.) for 20 min in PBS 1x for blocking non-specific labeling. The primary antibodies, namely Claudin-2 (1:100, Invitrogen), ZO-1, 1:100, (Invitrogen, see Table 1), and Lysozyme, 1:500, (Cell Signaling Technology, see Table 1), were diluted in 1.5% NDS and individually incubated ON at 4 °C. The next day, the sections were washed 3 × 2 min in PBS 1x and then incubated for 2 h at RT in the dark with the appropriate fluorochrome-conjugated secondary antibody: goat anti-rabbit Alexa Fluor 568, 1:333, (Invitrogen, see Table 1) or goat anti-rabbit Alexa Flour 488, 1:333, (Jackson Immuno Research, see Table 1), both diluted in 1.5% NDS. Tissue sections were washed in PBS 1x for 3 × 2 min and were incubated with a solution of Hoechst 1:1500 (Enzo life sciences, code: 52401) in PBS 1x for 10 min. The slices were washed in bi-distilled water 2 × 5 min and then mounted with an aqueous medium (FluoreGuard Mounting Medium, ScyTek Laboratories Inc.) [47].

Negative controls were performed by omitting the primary antibody to exclude unspecific immunolabeling. For antibodies information, see Table 1.

### 2.5. Quantitation and Statistical Analysis

Digital images of PAS- and A-Blue-stained structures were acquired with a microscope equipped with a video camera (Eclipse 200; Nikon Instruments, Tokyo, Japan) through a 4× objective. Tissue sections from all experimental groups were processed simultaneously to minimize staining variability. A region of interest (ROI) corresponding to the epithelial perimeter was manually defined, and the epithelium area was calculated as pixels (2 sections/animals; 5 animals/groups). Counting of PAS+ or A-Blue+ goblet cells was performed by two observers blinded to each other (2 sections/animal; 5 animals/groups). The ratio of A-Blue+/PAS+ was expressed as percentage. The PAS and A-Blue density was obtained from the ratio between the number of PAS+ or A-Blue+ goblet cells and the epithelium area calculated as pixels, expressed as percentage.

Aβ1–40/42 immunofluorescence labeling was visualized using a Leica Stellaris 5 confocal microscope (Leica Microsystems, Milan, Italy) equipped with a 63× oil immersion objective. Images were acquired as maximum intensity Z-projections of 6 consecutive confocal scans (0.8 µm z-step, 5 µm total thickness) with a frame size of 1024 × 1024 pixels. To minimize technical variability and ensure comparability among sample, all acquisition parameters, including laser power, gain, offset, and z-stack settings, were kept identical across all experimental groups. A total of 15 villi per animal for the ileum and 3 large villi per animal for the proximal colon were sampled. All quantitative analyses were performed using the ImageJ software. A quantitative assessment of the fluorescent labeling area was carried out in the different experimental groups. Quantitative analysis of Aβ1–40/42 immunofluorescence was performed using ImageJ software. Images containing the fluorescence signal of interest were converted to 8-bit greyscale before analysis. For each image, the mean intensity value of all grey pixels within the image was calculated. The threshold value was then defined as two times the mean intensity value and identically applied to all samples. Immunolabeling quantitation was performed within the selected ROI using the ratio between positive pixels above threshold and total pixels and expressed as percentage. The ROI corresponded to the area occupied by enterocytes and was selected according identical morphological criteria in all sections. All quantitative analyses were performed blinded to the experimental groups.

For DCLK1 and Claudin-2 immunofluorescence analysis, both in the ileal and proximal colon, one image of an entire section was acquired for each animal using a Axio Imager Microscope (Carl Zeiss Microscopy GmbH, Oberkochen, Germany), equipped with a 40× objective and a frame size of 1024 × 1024 pixels. For both markers, 6 animals per group were analyzed. Acquisition settings were maintained consistently among experimental groups.

Quantification of DCLK1 immunolabeling was obtained by counting the positive tuft cells for every animal of each group and comparing values between the groups. The density of tuft cells was calculated as the ratio between the number of positive cells and the ROI area (expressed in pixels) and reported as percentage. The ROI was manually defined using ImageJ software and selectively included the epithelial compartment. Cell counting and quantitative analyses were performed blinded to the experimental groups.

Quantification of the Claudin 2 immunolabeling was done using ROI corresponding to the entire epithelial perimeter designed manually by means of ImageJ software. The epithelium area was calculated as pixels (1 section/animal; 6 animals/groups). The quantification was expressed as a percentage of pixels positive to the immunolabeling over the total pixels of the ROI. Images were converted to a 8-bit greyscale before the analysis. For each image, the mean intensity value of grey pixels was calculated. The threshold value was defined as 2.5 times the mean intensity value and identically applied to all samples. All quantitative analysis was performed blinded to the experimental groups.

For ZO-1 immunofluorescence analysis in the ileal epithelium, images of 10 villi per animal (6 animals/group) were acquired using a Leica Stellaris 5 confocal microscope (Leica Microsystems, Milan, Italy) equipped with a 20× objective (1 µm z-step, 5 µm total thickness, frame size of 1024 × 1024 pixels). For the ascending colonic epithelium, 10 representative images of ZO-1 labeling per animal (5 animals/group) were captured using an Olympus BX63 fluorescence microscope (Tokyo, Japan) with a 20× objective. All acquisition parameters were kept constant across experimental groups to allow for consistent quantitative comparison.

Quantitation of ZO-1 immunofluorescence was performed using ImageJ software. Images were converted to grayscale prior analysis for each image, the mean intensity value of the entire image was calculated, and identical thresholding parameters were applied to all samples. The threshold value was then defined as 2.8 (ileum) and 1.8 (colon) times the mean intensity value and identically applied to all samples. Quantification was obtained from the ratio between positive pixels above threshold and total pixels in each ROI and expressed as a percentage. The epithelium was selected as the ROI. Quantitative analyses were performed blinded to the experimental groups.

For lysozyme-positive cell analysis in the ileum, images of the entire section (1 section/animal; 6 animals/group) were acquired using a Leica Stellaris 5 confocal microscope equipped with a 20× objective (frame size of 2048 × 2048 pixels). All acquisition parameters were maintained consistently across experimental groups to allow for consistent quantitative comparison. Paneth cells were identified based on lysozyme positivity and quantified by manual counting within the intestinal crypts using the multipoint tool of ImageJ software. The data were expressed as the number of lysozyme-positive cells per crypt. To perform the quantitation of lysozyme immunofluorescence corresponding to the secretion quantity of Paneth cells, the ROI was defined as the mucosa layer. This region was identified using ImageJ software by setting a threshold to distinguish the tissue from the background, followed by manually tracing a boundary along the luminal perimeter of the villi and the basal limit of the tunica mucosa. Images were converted to grayscale before analysis. For each image, the mean grey value of all the entire image was calculated. Quantification of lysozyme immunofluorescence was performed using ImageJ software and was obtained from the ratio between positive pixels above threshold and total pixels within the ROI and expressed as a percentage. The threshold value was defined as three times the mean intensity value calculated within the ROI and identically applied to all samples. Quantitative analyses were performed blinded to the experimental groups.

All the results are reported as the mean ± S.E.M. Statistical analysis was performed using *two-way* ANOVA, with disease status and treatment as the independent variables. Male and female were pooled for the analysis, and sex was not included as an additional factor in the statistical investigations. When ANOVA indicated significant differences, multiple comparisons among groups were carried out using the Newman–Keuls post hoc test [47]. Differences were considered statistically significant at *p* < 0.05. All statistical analyses and quantitative image analyses were performed using GraphPad PRISM v. 5 for Windows (GraphPad Software, San Diego, CA, USA) and ImageJ software (National Institute of Health, Bethesda, MD, USA; http://imagej.nih.gov/ij, accessed on 15 November 2020), respectively, using the same software versions for all samples and experimental groups [47].

## 3. Results

### 3.1. APP/PS1 Transgenic (Tg) Mice Show Alterations in the Lining Epithelium in the Ileum and Proximal Colon

**Figure 1:**
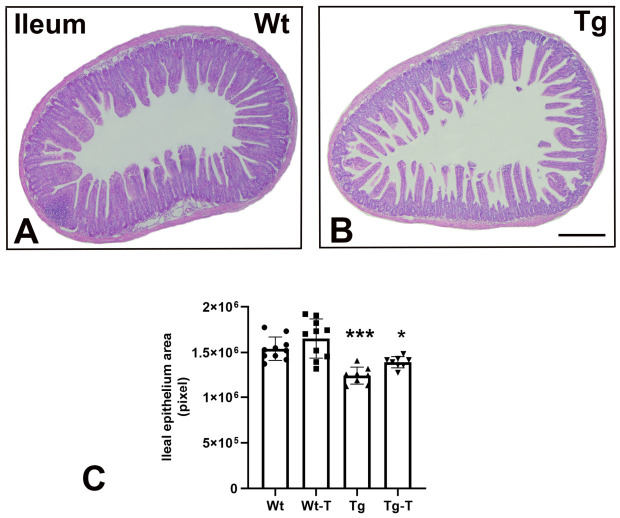
**Hematoxylin Eosin (H&E) staining of the ileum.** Representative image of the H&E staining in the ileum (**A**,**B**). Bar = 200 μm. Quantitation of the epithelial area (**C**) shows a significant reduction in Tg mice compared to all the other groups and a partial recovery in Tg-T mice. *** *p* < 0.001 Tg vs. Wt and Wt-T; * *p* < 0.05 Tg-T vs. Wt, Wt-T, and Tg. *Two-way* ANOVA; post hoc Newman–Keuls multiple comparison test (*N* = 5 animals/2 samples/animal).

In the ileum of Tg mice, the epithelial surface showed a significant reduction compared to the other groups (Figure 1).

**Figure 2:**
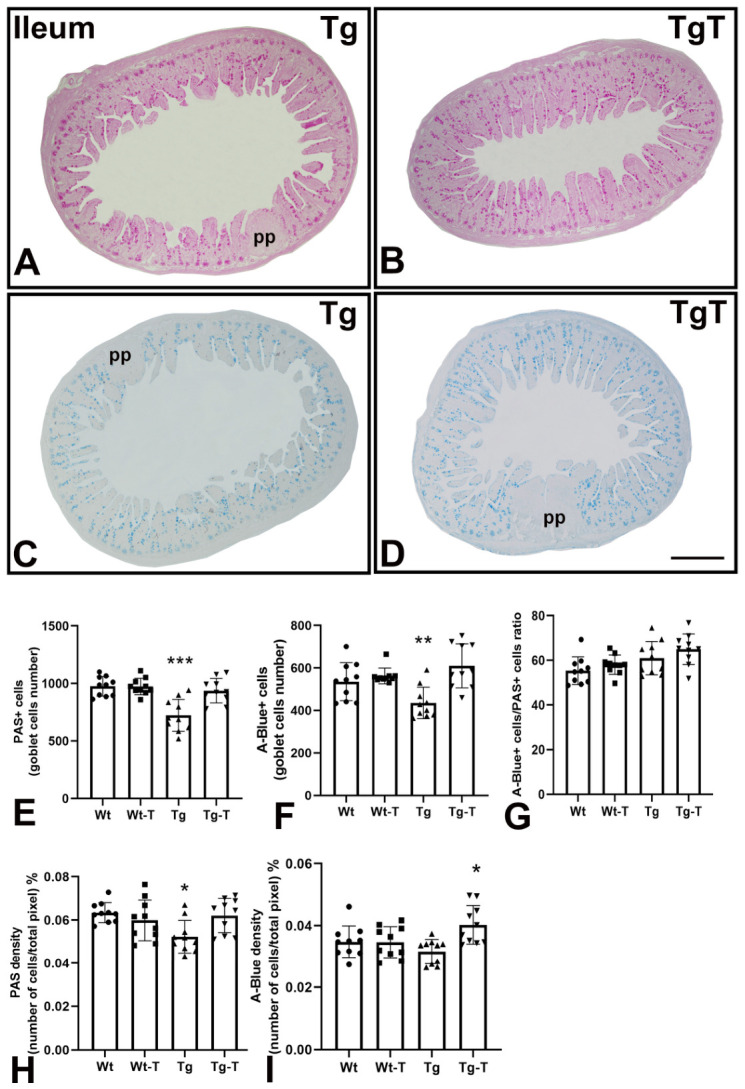
**Periodic Acid-Schiff (PAS) and Alcian Blue (A-Blue) staining in mouse ileum.** Representative images of PAS and A-Blue staining in the ileum of Tg and Tg-T mice. (**A**,**B**) The PAS staining (pink) is distributed in goblet cells along the villi and in the gland cells of the crypts. (**C**,**D**) A-Blue staining (light blue) is mainly distributed in the goblet cells, highlighting the acidic component of the mucus. Bar = 200 μm. Quantitation of both dyes shows that the number of PAS+ cells and of A-Blue+ cells is significantly decreased in Tg mice (**E**,**F**), while the A-Blue+ cells/PAS+ cells ratio is unchanged (**G**). The PAS+ cells density is also significantly decreased in Tg mice (**H**), while the A-Blue+ cells density shows a significant increase in the Tg-T mice (**I**). The synbiotic treatment prevents the impairment in mucus quantity and quality (**E**–**I**). *** *p* < 0.001 vs. the other groups; ** *p* < 0.01 vs. the other groups; * *p* < 0.05 vs. the other groups. *Two-way* ANOVA; post hoc Newman–Keuls multiple comparison test. pp = Peyer’s patches (*N* = 5 animals; 2 samples/animal).

PAS staining was used to label the mucus in the goblet cells and in the glandular crypts (Figure 2A,B), while A-Blue staining identified the acidic component of the mucus and was present mainly in the goblet cells along the lining epithelium (Figure 2C,D). The epithelial surface reduction (Figure 1A,B) was associated with a reduced mucus secretion and with a reduced number of PAS+ (Figure 2A,B) and A-Blue+ (Figure 2C,D) goblet cells. The decrease in mucous secretion observed in Tg mice (Figure 2E,F) seemed due to the reduced area of the epithelium surface, as the A-Blue+/PAS+ cells ratio was unchanged among the four groups of mice (Figure 2G). However, when the number of PAS+ goblet cells was expressed as density of PAS+ cells (number of cells/ROI expressed in pixels) on the epithelial surface, a significant decrease was observed in Tg mice, indicating a real reduction in mucus secretion in these mice (Figure 2H). On the contrary, the density of A-Blue+ goblet cells in Tg mice did not differ from the density in Wt and Wt-T mice, suggesting that the acidic component of the mucus was proportionally maintained (Figure 2I).

In the colon, previous data showed no change of the epithelial area but a significant decrease in mucus secretion in the same mouse strain at the same age (as previously reported [47]).

### 3.2. APP/PS1 Tg Mice Show Alterations of the Intestinal Barrier (IB) in the Ileum and Proximal Colon

**Figure 3:**
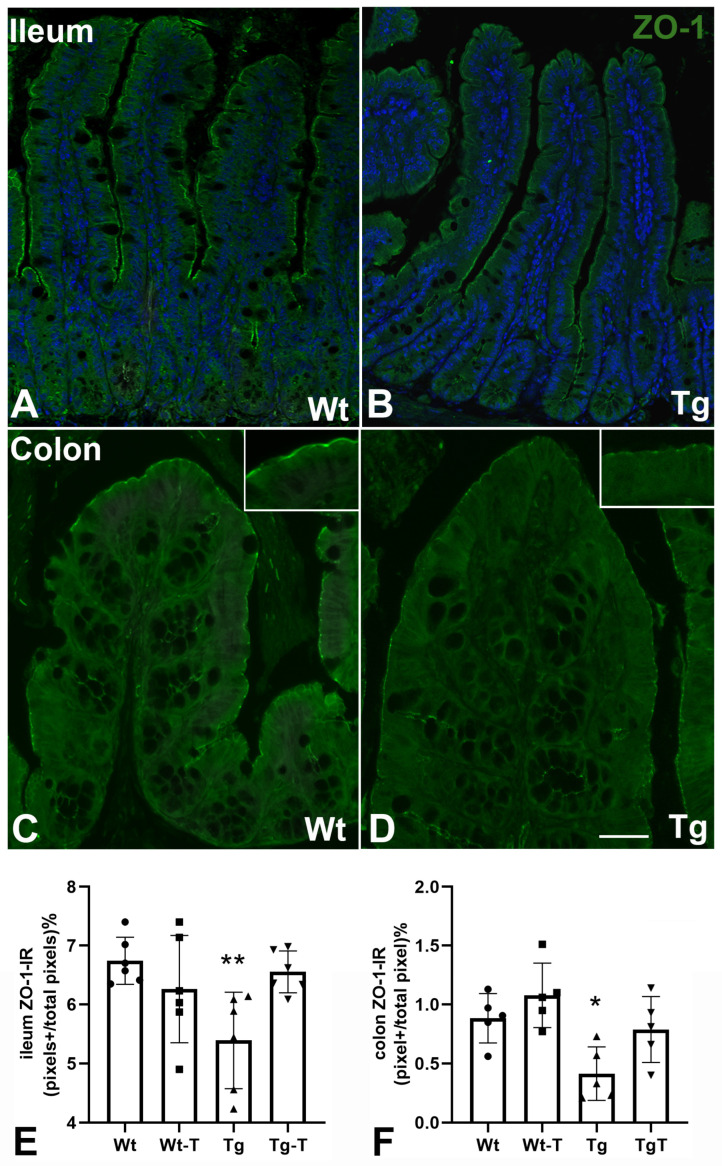
**ZO-1-immunoreactivity (IR)** (green) is located in the most apical portion of the lateral plasmalemma of ileal and colonic enterocytes (**A**–**D**); it appears as small dashes distributed along the cell perimeter (**A**–**D, inserts**). In blue: the nuclei. Bar = 20 μm. Quantitative analysis of ZO-1 labeling shows a significant decrease in both regions of Tg mice compared to the other groups (**E**,**F**). ** *p* < 0.01 and * *p* < 0.05 vs. the other groups. *Two-way* ANOVA; post hoc Newman–Keuls multiple comparison test. *N* = 6 animals.

The integrity of IB was evaluated by investigating the immunolabeling of ZO-1 and Claudin-2, proteins of the TJs. ZO-1 immunoreactivity (IR) was located in the most apical portion of the lateral plasmalemma of all groups (Figure 3A–D), appearing as small dashes distributed along the cell perimeter both in the ileum (Figure 3A,B) and ascending colon (Figure 3C,D, insert). Quantitation of ZO-1 labeling in the two regions showed a significant decrease in Tg mice compared to the other groups (Figure 3E,F).

In the ileum, this decrease was due to a real loss rather than a secondary effect of reduced area, as the immunolabeling was measured as a percentage of positive area within the selected villi (Figure 3E).

**Figure 4:**
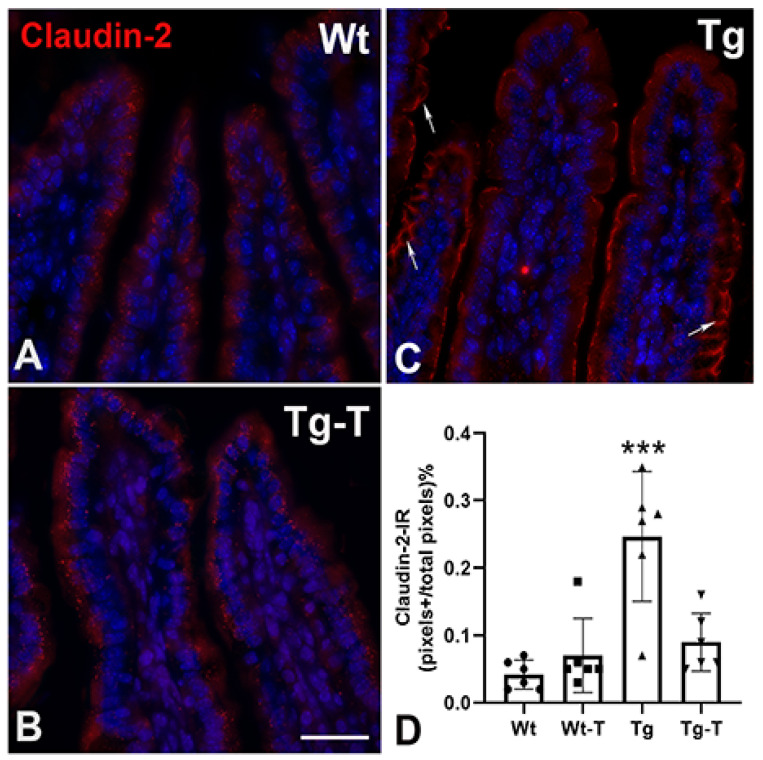
**Claudin-2 immunoreactivity (IR) in the ileum** is distributed in the upper third of the lateral membrane and has a punctate aspect in Wt (**A**) and Tg-T (**B**) mice, while it forms a thin and continuous line resembling a network in Tg (**C, arrows**) mice. Bar = 20 μm. Quantitative analysis of Claudin-2-IR shows a significant decrease in Tg mice compared to the other groups (**D**). *** *p* < 0.001 vs. the other groups. *Two-way* ANOVA; post hoc Newman–Keuls multiple comparison test. *N* = 6 animals.

Claudin-2 labeling was detected exclusively in the ileum. It was located in the upper third of the lateral membrane of the enterocytes and showed either a punctate pattern or a continuous profile resembling a thin network (Figure 4A,C). Quantitation of Claudin-2 labeling showed a significant increase in Tg mice compared to the other groups of mice (Figure 4D).

### 3.3. APP/PS1 Tg Mice Show Changes in the Innate and Adaptive Immunity in the Ileum but Not in the Proximal Colon

The immune response was assessed by studying the presence and distribution of tuft cells, which are considered mediators of the adaptive immune response, and the quantity and distribution of lysozyme, an antibacterial enzyme produced primarily by Paneth cells, which are part of the innate immune response and are located in the ileal crypts.

**Figure 5:**
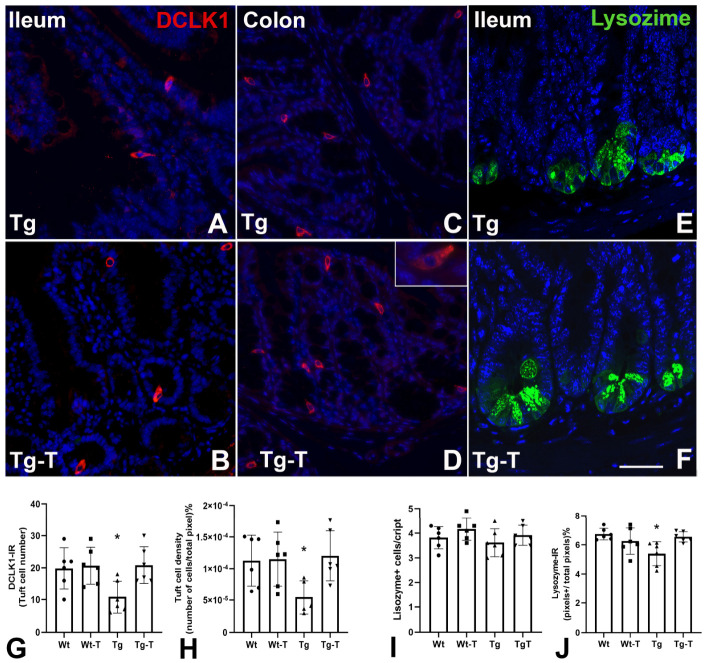
**DCLK1-immunoreactivity (IR) (red) in the ileum and colon and Lysozyme-IR (green) in the ileum.** DCLK1-IR is present in the epithelium of both regions in thin and elongated cells, whose apical portion opened into the lumen (**A**–**D**). The labeling is distributed throughout the cytoplasm up to the apical tuft (**Insert**). Lysozyme-IR is located in the Paneth cells of the ileum in granules of different size (**E**,**F**). In blue: the nuclei. Bar = 20 μm. Quantitative analysis of the DCLK1 labeling in the ileum shows a significant decrease both in the tuft cells number and tuft cells density in Tg mice (**G**,**H**). Quantitative analysis of the Lysozyme-IR showed no change in the number of positive cells (**I**), whereas the density of the enzyme was significantly decreased in Tg mice (**J**). * *p* < 0.05 vs. the other groups. *Two-way* ANOVA; post hoc Newman–Keuls multiple comparison test. *N* = 6 animals.

The tuft cells were stained with the DCLK1 antibody. DCLK1-positive cells were sparsely distributed throughout the lining epithelium in both the ileum (Figure 5A,B) and ascending colon (Figure 5C,D). The labeling was distributed throughout the cytoplasm up to the apical tuft (insert). In the ileum, the number and density (Figure 5G) of tuft cells were significantly reduced in Tg mice. In the ascending colon, unlike the ileum, these parameters did not differ between groups.

Lysozyme immunoreactivity (IR) was localized exclusively within the ileal crypts, specifically targeting the Paneth cell population (Figure 5E,F). The labeling was intense and located in granules of variable sizes predominantly distributed in the supranuclear compartment of the cytoplasm (Figure 5E,F).

Quantitation of lysozyme-IR showed no change in the number of positive cells (Figure 5I), whereas the density of the enzyme was significantly decreased in Tg mice (Figure 5J).

### 3.4. Prebiotics and Probiotics Treatment Protects the Integrity of Ileal and Colonic Epithelium and Intestinal Barrier (IB) in APP/PS1 Tg Mice

The long-term treatment (6 months) with probiotics and prebiotics was able to prevent most of the changes observed in the ileum and colon of APP/PS1 mice. Treatment partially protected the ileal epithelium since in Tg-T, the epithelium surface was significantly larger than in Tg but still smaller than in Wt and Wt-T mice (Figure 1). Conversely, the treatment not only completely prevented the loss of PAS+ goblet cells (Figure 2E,F) but caused a relative increase in their number, considering that the epithelial area in Tg-T mice was still reduced (compare Figure 1 vs. Figure 2H). Moreover, the number of A-Blue+ goblet cells was significantly increased in Tg-T compared to all the other groups, as shown when expressed as a density relative to the epithelium surface (compare Figure 1 vs. Figure 2I). These results agree with those previously reported in the colon in the same experimental conditions [47].

Both in the ileum and colon, prebiotics and probiotics protected against IB damage, preventing the reduction in ZO-1 (Figure 3E,F) and, in the ileum, the increase in Claudin-2 expression (Figure 4D). Finally, in the ileum, the treatment prevented the loss of tuft cells (Figure 5G) and the reduction in lysozyme content in the crypts (Figure 5J).

### 3.5. Probiotics and Prebiotics Treatment Increases the Expression of Aβ 1-40/42 in the Ileal and Colonic Epithelium of Wt-T and Tg-T Mice

**Figure 6:**
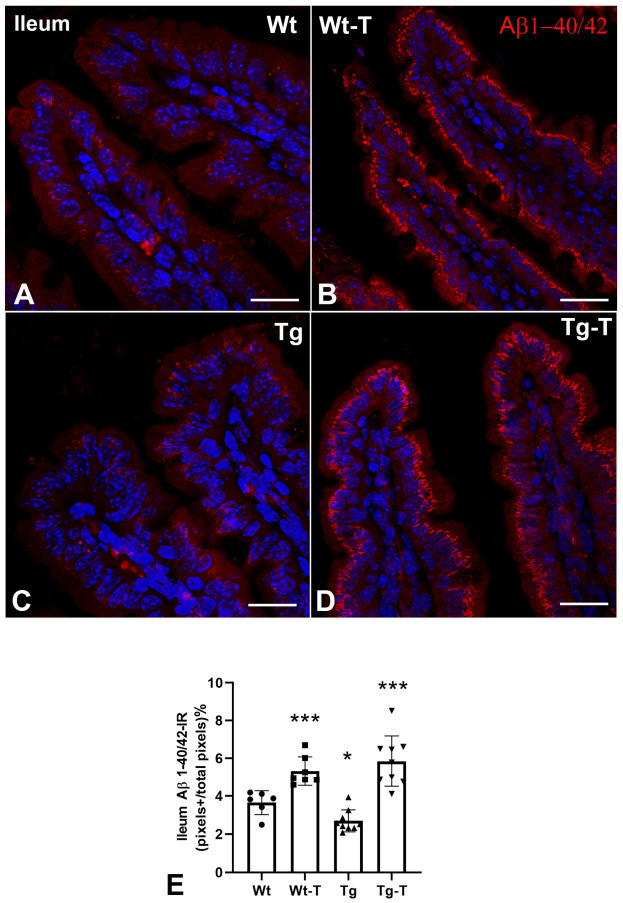
**Aβ1–40/42 immunoreactivity (IR) (red) in the enterocytes of mouse ileum.** Aβ1–40/42-IR was detected in the columnar cells of the lining epithelium; the labeling was located in the cytoplasm, immediately above the nucleus (**A**–**D**). In blue: the nuclei. Bar (**A**,**C**) = 20 μm; bar (**B**,**D**) = 40 μm. Quantitative analysis of the Aβ1–40/42 labeling shows a significant increase in both treated mouse groups vs. the untreated ones (**E**). The labeling intensity is significantly decreased in Tg mice compared to Wt (**E**). *** *p* < 0.001 vs. untreated mouse groups; * *p* < 0.05 vs. Wt. *Two-way* ANOVA post hoc Newman–Keuls multiple comparison test. Wt *n* = 6; Wt-T *n* = 6; Tg *n* = 9; Tg-T *n* = 6 animals.

**Figure 7:**
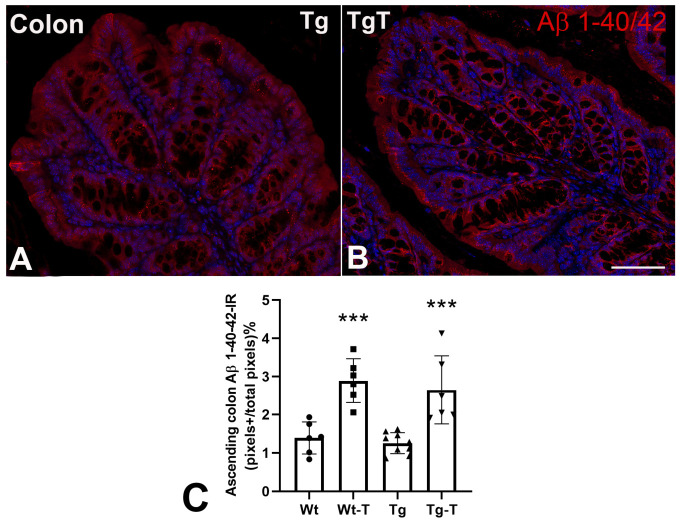
**Aβ1–40/42 immunoreactivity (IR) (red) in the enterocytes of mouse ascending colon.** Aβ1–40/42-IR is detected in the columnar cells of the lining epithelium, immediately above the nucleus (**A**,**B**). In blue: the nuclei. Bar = 40 μm. Quantitative analysis of the labeling shows a significant increase in both treated mouse groups vs. untreated groups (**C**). No difference was seen between Tg and Wt (**C**). *** *p* < 0.001 vs. untreated mouse groups. *Two-way* ANOVA post hoc Newman–Keuls multiple comparison test. Wt *n* = 6; Wt-T *n* = 6; Tg *n* = 9; Tg-T *n* = 6 animals.

To correlate the epithelial damages to the possible presence of/increase in the Aβ in the enterocytes, sections of ileum and colon of Wt, Wt-T, Tg, and Tg-T mice were immunostained with Aβ1–40/42 antibody. In all groups, the labeling of the amyloid peptides in the mucosa was present in the enterocytes, where it was concentrated in the intermediate portion of the cytoplasm, forming a semicircle immediately above the nucleus (Figure 6A–D; Figure 7A,B). Notably, the intensity of the labeling was greatly and significantly increased in Wt-T and Tg-T compared to Wt and Tg mice, both in the ileum (Figure 6E) and colon (Figure 7C). Furthermore, in the ileum, the Aβ expression was significantly reduced in Tg compared to Wt mice (Figure 6E).

## 4. Discussion

The intestinal epithelium represents the main and largest gateway for substances to enter the organism, and the associated MB is the major reservoir of bacteria in the human body. Extensive interaction between these bacteria and human digestive, immune, and nervous systems has been demonstrated [52]. The present study provides evidences that the integrity of the ileal and colonic epithelium of APP/PS1 mice is compromised, that the ileum is more damaged than the colon, and that treatment with synbiotics prevented most of the observed alterations.

In the ileum was observed (i) a significant reduction in epithelial surface area, associated with a marked decrease in mucus secretion; (ii) alterations in markers associated with IB function and with immune responses, such as decrease in ZO-1, an increase in Claudin-2 expression, and a reduction in the number of tuft cells and in lysozyme production by Paneth cells, respectively. In the colon, changes in IB-associated markers were also observed, although to a lesser extent compared to the ileum, while the tuft cell number was unchanged. In both gut regions, synbiotic treatment was able to prevent most of these changes. Finally, unexpectedly, the treatment was associated with a significant increase in Aβ1–40/42 expression in the enterocytes in both intestinal regions of Wt and Tg mice compared to the untreated mice.

The MB, the mucus layer, and the epithelium form an integrated system responsible for IB integrity and functionality. The disruption of any of these components can increase the permeability of the intestinal barrier, facilitating the passage of pathogens or endotoxins into the circulation, potentially reaching the CNS.

In this context, the host and microbiota are linked by a dynamic equilibrium in which the mucus layer plays a dual function, acting both as a physical barrier against luminal antigens and as a nutrient source for commensal bacteria. In turn, these microbes not only benefit from this environment but also contribute to host protection by limiting pathogen colonization and supporting intestinal immune homeostasis [53]. Alterations of this balance under dysbiotic conditions, as observed in Alzheimer patients and AD animal models, have been linked to shifts in microbial distribution including the accumulation of amyloid-producing bacteria such as *E. coli* within the luminal biofilm [54], whereas eubiotic conditions favor the presence of beneficial genera such as *Akkermansia* [55]. Consistent with these findings, we previously demonstrated an increase in the genus *Akkermansia* in the very same experimental cohort as the present study [47]. However, since no parallel microbiota or metabolome analyses were performed in this study, it is not possible to directly establish causal relationships between microbiota changes and intestinal alterations.

The mucus is secreted by goblet cells present both in the small intestine and in the colon and consists mainly of highly O-glycosylated glycoproteins layered along the gut wall [56]. However, important differences in the quality and quantity of this layer are present between the two gut regions. In the ileum, sialylated glycoproteins are predominant, while in the colon, these molecules contain fucose [57]. Moreover, while the colon has a thick and continuous layer, the ileum presents a patchy and more penetrable layer [58]. It is likely that these different features make the ileum more sensitive to perturbations in the microbial ecosystem [59].

The greater sensitivity of the ileum is further reinforced by the significant reduction in epithelial surface observed in Tg mice. This effect, restricted to the epithelium, may be related to the very short lifespan of these cells, whose turnover is the fastest of all epithelial cells, occurring in 3–5 days [60], making them particularly sensitive to changes in the local microenvironment. Accordingly, a positive relationship has been described between the main products of microbial metabolism, SCFAs, and intestinal epithelial proliferation [61,62]. In this context, studies on the pleiotropic hormone GLP-2 have indicated this hormone as the link between SCFA and stem cell activation [60]. The synbiotic treatment only partially prevented the loss of epithelial surface but completely prevented the decrease in mucus production in the ileum both in terms of goblet cell number and mucin content, in line with previous findings in the colon [47].

In addition to the mucus layer, TJs represent another fundamental component of IB: to control the translocation of luminal antigens into the systemic compartment [63]. TJs are highly dynamic complexes undergoing continuous remodeling, characterized by the constant turnover of proteins such as Claudins in response to different physiological stimuli or environmental and pathological insults [64,65]. In this context, several pathogens have developed mechanisms to disrupt this barrier by directly targeting TJ proteins or the cytoskeletal structures that maintain their stability [66]. In the present study, we observed alterations in both the TJ proteins investigated, namely ZO-1 and Claudin-2, in Tg mice. The ZO-1 expression was significantly reduced both in the ileum and colon of Tg mice compared to the other groups. A decrease in ZO-1 and other TJ proteins expression has been associated with neurodegenerative and metabolic disease [67,68]. At variance with ZO-1, Claudin-2 was found only in the ileum, and its expression was significantly increased in Tg mice compared to the other groups. Claudin-2 is a common component of the TJs and is extremely sensitive to local environmental changes; in particular, pathological conditions such as dysbiosis or inflammation cause increased expression of Claudin-2 that is associated with impaired IB integrity [69,70,71]. Thus, this latter datum suggests that TJ damage is greater in the ileum than in the colon. Overall, these findings are consistent with the hypothesis that the coexistence of dysbiosis and alterations in tight junctions (TJs) could constitute the ideal conditions for triggering local events responsible, over time, for neurodegeneration. In this scenario, these peripheral changes may precede central symptoms by years or even decades, signifying an early role of the gut–brain axis in disease progression [23,25,27].

The presence of a “leaky gut” [72], characterized by increased intestinal permeability, allows the passage of luminal antigens that activate both the innate and adaptive immune response locally and systemically [20,54,57,73]. We presently investigated two key cellular components of the intestinal epithelium involved in mucosa immune response: the tuft cells and the Paneth cells. The tuft cells are distributed along the axis of the villus, interspersed among the absorptive cells. They exhibit pear-shaped morphology, with a large basal portion and a narrow apex from which a tuft of microvilli projects into the lumen. Their presence and function have been linked to epithelial cell survival/self-renewal and mucosal healing, and they may function as sensors connecting the luminal microbiota to the host immune system [74,75], mediating type-2 mucosal immunity [76]. The Paneth cells are secretory cells located at the base of the small intestinal crypts, playing a critical role in innate immunity by secreting antimicrobial peptides such as lysozyme and contributing to maintenance of the IB by controlling the enteric bacteria [47,77]. In the ileum of APP/PS1 mice, the number of tuft cells significantly decreased, while no change was detected in the colon. Conversely, the Paneth cells number was unchanged, but a significant reduction in lysozyme production was detected. These results agree with previous studies reporting a constant involvement of intestinal innate and adaptive immunity in AD patients [73] and AD animal models [54], and they represent, to our knowledge, the first evidence of changes in tuft cell number and lysozyme content in the ileum of an AD mouse model. Both parameters seem to be closely related to the MB. Lysozyme is secreted in response to bacterial antigens entering the intestinal wall in the presence of a leaky barrier [72]. Therefore, the observed reduction in lysozyme content within Paneth cells could reflect the secretion and subsequent reduction in intracellular stores, potentially triggered by the translocation of luminal antigens in Tg mouse epithelial cells. The role of tuft cells may be more complex. Treatments with probiotics and zinc increase the number of tuft cells in the small intestine, highlighting the role of nutrients in epithelial integrity [78,79]. Furthermore, Chen et al. described a relationship between tuft cell loss and dysbiosis and found a correlation between these conditions and cognitive impairment [80]. Tuft cells have a pivotal role in epithelial cell proliferation and differentiation [79,81]. Based on this, we hypothesize that the decrease in tuft cell number and the decrease in epithelial surface area in the ileum of Tg mice may be correlated. Finally, the finding by Edens-Valentine et al. [82] that *Akkermansia muciniphila* treatment increased the number of tuft cells in the ileum could explain the beneficial effects of the synbiotic treatment on these cells, in line with our previous report on an increase in this bacterium in the same transgenic strain subjected to the same treatment [47].

The mechanisms underlying the beneficial effects of the synbiotic treatment remain to be fully clarified, and the present study does not allow discrimination between the relative contribution of the probiotic strains, the prebiotic components, or microbiota metabolites. As mentioned above, *Lactobacilli* contribute both directly [51] and indirectly to SCFA production through cross-feeding mechanisms, for instance, by producing lactate that can be further transformed into butyrate by other commensal bacteria [50]. In addition, the prebiotics administered in this study serve as substrates for a broader range of gut microbial species, thereby promoting SCFA production and contributing to a more balanced gut microbial ecosystem [83,84].

SCFAs play a fundamental role in maintaining the overall health of the organism. After being produced in the gut lumen, they can cross both the intestinal and brain barriers [50,83], exerting their effects systemically. At the intestinal level, SCFAs enhance TJ protein expression, which strengthens epithelial barrier maintenance [83] and exerts anti-inflammatory effects by promoting T-cell differentiation toward anti-inflammatory phenotypes and decreasing the production of pro-inflammatory cytokines [85]. Moreover, SCFA receptors are expressed on immune cells, and SCFAs can modulate immune signaling pathways that influence brain function, potentially exerting protective effects [86]. In addition, SCFAs may indirectly modulate aryl hydrocarbon receptors (AHR) signaling, whose activation decreases gut inflammation and reduces both gut and BBB permeability [50].

The final step in our investigation was to assess whether IB damage could be associated with increased Aβ1–40/42 expression in enterocytes as a consequence of dysbiosis. The results obtained were largely unexpected. We observed that untreated Wt and Tg mice expressed low levels of Aβ1–40/42 in the ileal and colonic epithelium, in agreement with data published by Galloway in control mice. However, in the ileal epithelium of Tg mice, which appeared to be the region most affected by the disease, Aβ1–40/42 expression levels were even lower than in Wt mice. Finally, and most surprisingly, Aβ1–40/42 expression increased significantly in the ileal and colonic enterocytes of all treated mice regardless of their genetic background. To our knowledge, this finding has never been reported before and deserves in-depth discussion.

Before entering the discussion, it is important to clarify the antibody-related aspects underlying Aβ detection in the gut. Among the antibodies tested (see Table 1), only the Aβ1–40/42 antibody was able to reliably label gut tissue when a specific protocol adapted from Galloway et al. [26,27,87] (see Section 2) was applied. This antibody recognized both Aβ peptides 1–40 and 1–42 and may also detect APP, according to the manufacturer’s datasheet. Moreover, all the antibodies tested (Aβ1–40/42, Aβ1–16, and Aβ1–42) were able to label the characteristic amyloid plaques in the brain sections, confirming their suitability for Aβ detection. Plaques were evident in Tg mice and reduced in size and number following treatment (personal observations). Importantly, these results agree with those obtained using the Aβ1–16 antibody in previous studies [48,49], where it was shown to reliably label amyloid plaques, further supporting the goodness of the staining.

For all these reasons, we considered (i) the enterocytes labeling to be reliable; (ii) the increased Aβ1–40/42 immunoreactivity to be associated with synbiotic-induced microbiota changes; that (iii) this increase may reflect adaptive or protective processes given its association with the improvement or complete recovery in the numerous parameters measured in treated Tg, both in the present and in the previous studies [47,48,49]. However, the biological significance of this finding remains unclear and should be interpreted cautiously since no direct analyses of APP processing, Aβ degradation pathways, or systemic Aβ dynamics were performed.

Using the same antibody and experimental procedure, Aβ1–40/42 labeling has previously been reported in mouse ileal enterocytes in a different animal model, and its intensity has been shown to be modulated by diet and fasting [26,27]. In these studies, the authors proposed that intracellular Aβ1–40/42 is synthesized locally in association with chylomicron biogenesis through lipid incorporation, particularly saturated fatty acids and cholesterol, facilitating their metabolism processing and solubility. In turn, increased lipidation of circulating lipoproteins has been proposed to influence Aβ trafficking, potentially including peripheral Aβ derived from the brain [27,88].

In light of this information, the increased Aβ1–40/42 immunoreactivity observed in enterocytes of treated mice may represent a local response potentially involved in facilitating the incorporation of cholesterol and saturated fatty acids into chylomicrons and lipoproteins. This process could hypothetically contribute to modulating systemic lipid and amyloid homeostasis and may potentially limit peripheral Aβ availability and brain exposure [18]. Moreover, increased lipoprotein lipidation may be involved in peripheral clearance pathways of Aβ1–40/42, including transport between the brain, blood, and peripheral organs such as the liver and intestine [42,88]. In fact, more than 95% of circulating Aβ1–40/42 is bound to lipoproteins [88]. In particular, apoA-I interacts with Aβ, preventing its aggregation [89], and apoA-I-HDL complex has a high tendency to bind to Aβ and clear it through the BBB [88]. However, the present data do not allow determination of whether enterocytes actively contribute to Aβ production, processing, transport, or clearance, and additional evidence would be required to support our hypothesis, which is still partial and speculative. In particular, additional analyses addressing APP-processing enzymes, Aβ-degrading pathways, circulating or luminal Aβ levels, and the relationship between intestinal and brain Aβ burden would be necessary to clarify the potential role of enterocytes in Aβ handling.

Notably, in recent years, the framework for understanding the physiological and pathological roles of Aβ40–42 in the neuronal system has become increasingly broad, and the classic amyloid cascade hypothesis has been re-evaluated for several reasons: The first reason is the extremely disappointing results obtained following treatments targeting the Aβ peptide or the enzymes responsible for its production [1,17,18]. The second is that studies on the formation and localization of Aβ have revealed not only a greater number of functions of this peptide than previously assumed in neuronal physiology but have also highlighted its dual antimicrobial behavior, leading to the hypothesis that AD may involve immune-related mechanisms [90]. Third, heterogeneous findings have been reported regarding Aβ levels in blood, although recent ultrasensitive assays have improved their diagnostic reliability [25,91], while reduced cerebrospinal fluid (CSF) Aβ42 in AD patients is widely recognized as a robust biomarker of AD pathology [92,93]. Finally, alternative hypotheses have questioned the central role of Aβ aggregation in AD progression, including the so-called Beta-Amyloid Dysfunction (BAD) hypothesis [1].

## 5. Conclusions

In conclusion, although our study does not directly evaluate functional parameters such as intestinal permeability or its modifying effects on neurodegeneration, it demonstrates that long-term symbiotic treatment, initiated at a young age, exerts a protective effect on the epithelium, counteracting the alterations observed in our APP/PS1 model. The unexpected upregulation of Aβ1–40/42 in enterocytes of synbiotic-treated mice, rather than reflecting a pathological process, suggests the existence of a novel synbiotic-induced APP-derived peptide modulation pathway. Overall, these findings support the hypothesis that the microbiota–gut–brain axis may contribute to systemic peptide homeostasis and highlight the potential of microbiota-targeted strategies as supportive interventions and a potential avenue for further research on neurodegeneration.

## Figures and Tables

**Table 1:** Primary and secondary antibodies used for immunohistochemistry.

Target	Antigen	Supplier	Catalog #	Antibody	Host	Usage	Dilution
β-Amyloid	β-Amyloid 1–40/42	Chemicon (Saint Lousi, MO, USA)	AB5076	Polyclonal	Rb	Primary	1:200
β-Amyloid	β-Amyloid 1–16	Biolegend (Dedham, MA, USA)	39320-200	Monoclonal	Ms	Primary	1:200
β-Amyloid	β-Amyloid 1–42	Cell Signalling (Danvers, MA, USA)	24090	Monoclonal	Rb	Primary	1:200
Paneth cells	Lysozime C	Cell Signalling	60487	Monoclonal	Rb	Primary	1:500
Tuft cells	Doublecortin-like kinase 1	Abcam (Cambridge, UK)	Ab-31704	Polyclonal	Rb	Primary	1:100
ZO-1	Zonula occludens 1	Invitrogen (Waltham, MA, USA)	40-2200	Polyclonal	Rb	Primary	1:100
Claudin-2	Claudin-2	Invitrogen	51-6100	Polyclonal	Rb	Primary	1:100
Goat FC	Goat FC	Thermo Fisher (Waltham, MA, USA)	A-11055	Polyclonal	Dn	Secondary Alexa Fluor 488	1:333
Rat FC	Rat FC	Thermo Fisher	A-11006	Polyclonal	Gt	Secondary Alexa Fluor 488	1:333
Mouse	Mouse FC	Thermo Fisher	A31570	Polyclonal	Dn	Secondary Alexa Flour 555	1:400
Rabbit FC	Rabbit FC	Jackson Immuno Research (Ely, UK)	111-575-144	Polyclonal	Gt	Secondary Alexa Fluor 568	1:333/ 1:100
Rabbit FC	Rabbit FC	Jackson Immuno Research	111-547-003	Polyclonal	Gt	Secondary Alexa Fluor 488	1:333

## Data Availability

The raw data supporting the conclusions of this article will be made available by the authors on request.
